# Survey of Advance Care Planning Practices in Elderly COVID-19 Patients: A Multicenter Questionnaire Survey

**DOI:** 10.7759/cureus.39663

**Published:** 2023-05-29

**Authors:** Ryosuke Morio, Takahiro Takazono, Ryotaro Kubo, Koki Fukushima, Naofumi Suyama, Takuya Izumikawa, Koichi Izumikawa, Katsunori Yanagihara, Hiroshi Mukae

**Affiliations:** 1 Department of Internal Medicine, Izumikawa Hospital, Nagasaki, JPN; 2 Department of Respiratory Medicine, Nagasaki University Hospital, Nagasaki, JPN; 3 Department of Infection Control and Education Center, Nagasaki University Hospital, Nagasaki, JPN; 4 Department of Laboratory Medicine, Nagasaki University Hospital, Nagasaki, JPN; 5 Department of Respiratory Medicine, Nagasaki University Graduate School of Biomedical Sciences, Nagasaki, JPN

**Keywords:** healthcare provider, elderly patients, end-of-life care, coronavirus disease (covid-19), advance care planning (acp)

## Abstract

Objective

Advance care planning (ACP) is a process in which the patient and family discuss end-of-life care in advance with healthcare providers in case decisional ability declines. Due to the rapid deterioration of symptoms and isolation for treatment, it is not easy for COVID-19 patients to discuss their end-of-life care with families and medical staff. We conducted a questionnaire survey to assess the current situation of ACP practices in hospitalized COVID-19 patients.

Materials and methods

Multicenter questionnaire surveys of hospitalized COVID-19 patients aged ≥60 years between January 2021 and August 2022 were conducted in two hospitals. The questionnaires assessed whether patients had discussed end-of-life medical treatment with their families and family physicians at the time of admission and their preferences for end-of-life medical treatments.

Results

A total of 109 patients aged 60-99 years (median 75.0 years) were enrolled. Only eight patients (7.3%) had practiced ACP at the time of admission. Age was a significant factor related to ACP practices (p=0.035). Although there was no significant difference between the ACP and non-ACP practiced groups for each end-of-life care, all eight patients in the ACP-practiced group were able to make decisions for all the end-of-life medical treatment, while 40 patients (33.0%) in the non-ACP-practiced group did not, showing a significant difference (p=0.026).

Conclusion

In hospitalized COVID-19 patients, the rate of ACP practice was as low as 7.3%. Awareness of ACP practice is necessary, especially for elderly patients with underlying diseases.

## Introduction

COVID-19 is an infectious disease caused by SARS-CoV-2. It first emerged in Wuhan City, Hubei Province, China, in December 2019 and rapidly spread worldwide. As of January 25th, 2023, it had infected 32,251,054 people and caused 66,297 deaths in Japan [1]. The Omicron variant causes less severe pneumonia than the prior strains, and the rate of hospital admissions and ICU stays has decreased [2]. Moreover, vaccination has reduced the incidence of COVID-19 and mortality [3]. However, due to an increase in the number of infected patients, 1,342 deaths were reported in the seventh wave of COVID-19 in Japan, 71.3% of whom were aged 80 years or older, and 29.5% were reported to have died from causes other than COVID-19 [4]. Although the overall mortality rate is decreasing, elderly COVID-19 patients may still die during hospitalization, and the risk of death should be explained to patients and their families at the time of admission. Advance care planning (ACP) is a process in which the patient and family discuss end-of-life care and nursing care in advance with healthcare providers in case the patient’s decisional ability declines [5]. A survey conducted in Japan using a stratified two-stage random sampling of men and women aged 20 years or older reported that only 2.7% of respondents answered that they had made a decision about their preferred medical treatment in advance through discussions with their families and family physicians [6]. In addition, in a retrospective cohort study of 379 patients with dementia in Japan [7], ACP was practiced in 23.2% of patients. A total of 49.8% of patients who had discussions with their families about end-of-life care started these discussions after the diagnosis of dementia, and 11.6% of patients started before the diagnosis. In a survey of end-stage pneumonia in Japan among residents of long-term care hospitals and nursing homes, ACP was practiced in 16.2% of the patients [8]. Although ACP has recently gained importance in clinical practice, not many patients have practiced ACP. COVID-19 differs from chronic diseases due to the rapid deterioration of symptoms and isolation requirements. Further, it is difficult for patients to discuss end-of-life care with family members and healthcare providers [9]. Therefore, we conducted a questionnaire survey on the current situation of ACP practice in COVID-19 patients requiring hospitalization.

## Materials and methods

Questionnaire surveys were conducted for hospitalized COVID-19 patients aged 60 years or older. These were carried out at Nagasaki University Hospital, an academic medical center with 871 beds, and Izumikawa Hospital, Eiwakai Medical Corporation, a rural acute care hospital with 120 beds. The surveys occurred between January 4, 2021, and August 31, 2022. The attending doctors performed the questionnaire. We assessed whether the patients had discussed end-of-life medical treatment with their families and medical care providers at the time of admission by using the question sheet and evaluated their preferences for end-of-life medical treatment, such as cardiac massage, intratracheal intubation, tracheostomy, and parenteral nutrition. Similar questionnaires were also administered to the patients' key family members at the time of admission, either in person or by telephone. Only family members were interviewed when the attending physician determined that the patient could not make decisions regarding treatment due to cognitive function and medical conditions. We defined those who had discussed end-of-life medical care with both the family and family physician at the time of admission as practicing ACP. Patient information on age, sex, performance status (PS), decisional ability, Japanese definition of COVID-19 severity [10] at the time of admission, and underlying disease was collected from the patients' medical records. 

Next, we evaluated the association between various characteristics and ACP practice. These characteristics included sex, age, performance status (PS) on a scale of 0-2 and 3-4, decision-making ability, severity at the time of admission, and the location of prior residence (home or nursing home). We also considered underlying diseases that could affect COVID-19 severity, such as diabetes mellitus, chronic respiratory diseases (e.g., chronic obstructive pulmonary disease, bronchial asthma), cardiovascular diseases (e.g., ischemic heart disease, chronic heart failure), cerebrovascular diseases (e.g., cerebral infarction, cerebral hemorrhage), malignancies, chronic liver diseases, chronic kidney diseases, and autoimmune diseases (e.g., rheumatoid arthritis) [11]. Wishes regarding emergency treatments, if they were end-of-life conditions, such as chest compressions, tracheal intubation, tracheotomy, and central venous nutrition, were collected at the time of admission. 

This study was approved by the Nagasaki University School of Medicine Research Ethics Committee (approval number: 2210171). This study was a retrospective cohort study, so written informed consent was not obtained. However, after approval from the ethics committee of our institute, we have shown the document "Disclosure of Information on Clinical Research" on our homepage to give the subjects the opportunity to declare their will not to participate in this study.

Statistical analysis

Summaries of observations from categorical variables were provided as frequencies and continuous variables were presented as medians and interquartile ranges. Associations between variables were analyzed using Fisher's exact test or the Mann-Whitney test. Statistical analyses were performed using GraphPad Prism version 9.

## Results

Patient characteristics

The patient characteristics are listed in Table 1. There were 109 patients in total, aged 60-99 years (median 75.0 years); 67 patients (61.5%) were male, 28 patients (25.7%) had a PS of 3 or above, 81 patients (74.3%) had decisional ability, 65 patients (59.6%) had moderate COVID-19 or above at the time of admission, and 76 patients (69.7%) had at least one underlying disease related to the severity risk of COVID-19.

**Table 1:** Patient characteristics

Patient characteristics (n=109)	
Sex (M/F)	67/42
Age (range)	77.32 (60-99)
Performance status	
0 (%)	40 (36.7)
1 (%)	32 (29.4)
2 (%)	9 (8.3)
3 (%)	15 (13.8)
4 (%)	13 (11.9)
Decisional ability (presence/absence) *	81/28
Severity of COVID-19	
Mild (%)	44 (40.4)
Moderate Ⅰ(%)	31 (28.4)
Moderate Ⅱ(%)	34 (31.2)
Severe (%)	0 (0.0)
Hospitalization	
From home (%)	94 (86.2)
From a nursing home (%)	15 (13.8)
Underlying diseases	
Diabetes mellitus (%)	26 (23.9)
Chronic respiratory disease (%)	16 (14.7)
Cardiovascular disease (%)	21 (19.3)
Cerebrovascular disease (%)	15 (13.8)
Malignancy (%)	16 (14.7)
Chronic liver disease	2 (1.8)
Chronic kidney disease (%)	21 (19.3)
Autoimmune disease (%)	6 (5.6)
None (%)	33 (30.3)

Results

We found that only eight patients (7.3%) had practiced ACP at the time of admission. Table 2 shows a comparison of ACP practice and their characteristics; only the characteristic, age, was significantly different (p=0.035) in the ACP-practiced group aged 72-99 years (median 85.5 years) compared to those in the non-ACP-practiced group (60-97 years (median 74.0 years)). However, sex, PS, decisional ability, the severity of COVID-19 at the time of admission, hospitalization from home or nursing home, and underlying disease related to the severity risk of COVID-19 (diabetes mellitus, chronic respiratory disease, cardiovascular disease, cerebrovascular disease, malignancy, chronic liver disease, chronic kidney disease, autoimmune disease) were not significantly different.

**Table 2:** Comparison of characteristics in ACP and non-ACP groups. ACP: Advance care planning; PS: Performance status.

	ACP group	Non-ACP group	
Sex			
Male (%)	6 (75.0)	61 (60.4)	p = 0.708
Female (%)	2 (25.0)	40 (39.6)	
Age			
Range (mean)	72-99 (85.5)	60-97 (74.0)	p = 0.035
PS			
0-2 (%)	4 (50.0)	77 (76.2)	p = 0.199
3-4 (%)	4 (50.0)	24 (23.8)	
Decisional ability			
Presence (%)	4 (50.0)	77 (76.2)	p = 0.199
Absence (%)	4 (50.0)	24 (23.8)	
Severity of COVID-19			
Mild (%)	4 (50.0)	40 (39.6)	p = 0.712
Moderate (%)	4 (50.0)	61 (60.4)	
Hospitalization from			
Home (%)	7 (87.5)	87 (86.1)	p >0.999
Nursing home (%)	1 (12.5)	14 (13.9)	
Underlying diseases			
One or more	4 (50.0)	71 (70.3)	p = 0.253
None	4 (50.0)	30 (29.7)	

Table 3 shows whether patients in the ACP and non-ACP-practiced groups desired end-of-life care. Although there was no significant difference between the ACP and non-ACP-practiced groups for each treatment, eight patients (100%) in the ACP-practiced group were able to make decisions for all treatments. In comparison, 61 patients (60.4%) in the non-ACP-practiced group were able to make decisions, showing a significant difference (p=0.026). In the non-ACP-practiced group, where both the patient and the family could express their preferred care (47 cases), we also assessed any discrepancy between the patient's and the family's wishes regarding the desired treatment. We found divergent wishes in as many as 34% (16/47) of these cases, as shown in Table 4.

**Table 3:** The number of patients who were able to make decisions for treatments between ACP and non-ACP groups. ACP: Advance care planning.

	ACP group	Non-ACP group	
Cardiac massage (%)	8 (100)	77 (76.2)	p = 0.196
Intubation (%)	8 (100)	72 (71.3)	p = 0.106
Tracheostomy (%)	8 (100)	71 (70.3)	p = 0.104
Parenteral nutrition (%)	8 (100)	69 (68.3)	p = 0.102
All treatments (%)	8 (100)	61 (60.4)	p = 0.026

**Table 4:** The divergence between patient and family members regarding requesting procedures.

	Patient requested more invasive procedures	Family members requested more invasive procedures	Patient could not decide	Family members could not decide	Both of them requested the same procedures	Both of them could not decide
Chest compression	0	2	4	7	3	0
Intubation	1	2	2	7	2	2
Tracheotomy	0	2	3	6	1	4
Central venous nutrition	1	1	2	7	3	2

## Discussion

In the present study, only 7.3% of hospitalized COVID-19 patients had practiced ACP effectively with their families and medical care providers. ACP was practiced in elderly patients; however, PS, decisional ability, hospitalization from home or nursing home, and underlying disease related to the severity of COVID-19 were not significantly associated with the rate of ACP practice. In this study, more patients in the ACP-practiced group were able to make decisions about end-of-life care at the time of admission, suggesting that advanced decision-making is essential. ACP is often initiated with the onset of an underlying disease such as dementia [7]. The lower rate of patients with underlying diseases in our study might be the reason why the rate of ACP practice was lower than that in the previous report. However, decisional ability declines with the progression of dementia, and we believe it is more appropriate to initiate ACP before losing decision-making ability. Although few studies have investigated the rate of ACP practice at the time of admission in COVID-19 patients, a report from the United States evaluated ACP practice rates by race among COVID-19 patients in a multicenter, retrospective cohort study [12] and reported that white/hispanic/black patients had ACP practice rates of 12%, 12%, and 11%, respectively, with no significant differences and low rates of practice. A retrospective cohort study in the US compared the rate of ACP practice in the positive and negative SARS-CoV-2 PCR test groups and found that the positive group had a lower rate (12.9%) than the negative group (23.8%) at the time of admission. However, the results were not statistically significant [13]. The low rate of ACP practice observed in our study may be due to insufficient awareness of the concept of ACP in Japan. This could be related to the cultural tendency in Japan for thoughts and wills to be shared with others implicitly, with less reliance on explicit language. This is reflected in the Japanese concept of 'Ishin denshin,' which translates to 'heart-to-heart communication' [14]. Thus, patients may expect their family members to understand their intentions without explicit discussion." In addition, in examining each type of medical facility in Japan, 72.7% of hospitals, 77.2% of clinics, 59.9% of elderly care welfare facilities, and 65.7% of elderly nursing care facilities did not practice ACP [6]. To promote ACP practice, providing information and skills necessary for ACP to family physicians and other healthcare providers is essential. A project to provide online training on ACP in eight UK nursing homes during the COVID-19 epidemic has been reported [15]. This activity may be applied in Japan, and it may contribute to the dissemination of ACP practices. 

A multicenter study in US nursing homes [16] reported that the proportion of those who did not resuscitate (DNR) and did not hospitalize (DNH) increased post-ACP compared to pre-ACP. In a survey of patients with end-stage pneumonia in Japanese convalescent hospitals and nursing homes [8], it was reported that family members significantly preferred highly invasive procedures more than physicians. This suggests a gap between physicians, patients, and family members who prefer invasive treatment. This gap may be due to insufficient information regarding the details of medical procedures being shared with the family. It is also reported that the proportion of hospital deaths in Japan is 78.7%, much higher than that in Western countries (Netherlands 29.1%, Switzerland 38.2%, US 43.0%, England 49.1%, France 59.0%) [17]. In Japan, one of the factors contributing to excessive treatment is that medical costs are lower than those in other developed countries due to the universal health insurance system. In addition, the patients tend to receive more aggressive therapy due to the family's poor recognition of the patient's condition. As in previous reports, ACP allows a smooth transition to end-of-life care without overly invasive medical treatment beyond the patient's wishes. As COVID-19 is a potentially sudden and life-threatening infectious disease, patients should prepare for it in advance, even patients without major underlying medical diseases. In patients with COVID-19, ACP is of great importance, as it plays a major role in the ability to make calm decisions regarding treatment and in providing desired care for each patient and family. Our study is important because we provided the current status of ACP practice in Japan during the COVID-19 epidemic, and ACP can help patients make decisions about end-of-life care. The limitations of our study are as follows: Due to the small sample size (109 patients), especially the reduced number in the ACP group, we did not find sufficiently significant differences between the practice of ACP and the characteristics. In addition, the study period included the epidemic of the Delta variant with high lethality and the Omicron variant with low lethality. However, we believe that the findings obtained in our study will help Japanese clinicians initiate ACP.

## Conclusions

In our survey, 7/8 of the patients in the ACP-practiced group and 36/101 in the non-ACP-practiced group did not want life-prolonging treatment. It may be difficult for patients to express their wishes after developing COVID-19, and isolation may prevent them from fully discussing end-of-life care with key family members and healthcare providers. Especially in elderly patients with underlying diseases associated with the severity of COVID-19, ACP should be initiated when they are in good health. Therefore, further large-scale surveys are needed.

## Appendices

Supplementary information COVID-19 Pre-admission Instructions (for patients)

Have you ever discussed life-prolonging treatment with your family members and family physicians? fully discussed /only slightly discussed /never discussed at all Have you ever discussed with your families whether you would receive life-prolonging treatment? fully discussed / only slightly discussed /never discussed at all Do you wish to receive cardiac massage in the event of an emergency? yes /no /can't decide now Do you wish to receive mechanical ventilator management in case of a respiratory emergency? yes /no /can't decide now Do you wish to undergo a tracheotomy? Yes /No /can't decide now Do you wish to receive parenteral nutrition therapy? Yes /No /can't decide now

COVID-19 Pre-admission Instructions (for family members) What is your relationship with the patient? spouse /father /mother /sibling /children /grandchildren /other relative /acquaintance How old are you? 10s /20s /30s /40s /50s /60s /70s /80 years old or older How is your status related to SARS-CoV-2? non-infected /close contact person /infected Have you ever discussed the patient's life-prolonging treatment with him or her and family physicians? fully discussed /only slightly discussed /never discussed at all Have you ever discussed with him or her whether the patient would receive life-prolonging treatment? fully discussed / only slightly discussed /never discussed at all Do you wish the patient to receive cardiac massage in the event of an emergency? yes /no /can't decide now Do you wish the patient to receive mechanical ventilator management in case of a respiratory emergency? yes /no /can't decide now Do you wish the patient to undergo tracheotomy? Yes /No /can't decide now Do you wish the patient to receive parenteral nutrition therapy? Yes /No /can't decide now

## References

[1] (2023). Outbreak of COVID-19. https://www.mhlw.go.jp/stf/covid-19/kokunainohasseijoukyou.html.

[2] Maslo C, Friedland R, Toubkin M, Laubscher A, Akaloo T, Kama B (2022). Characteristics and outcomes of hospitalized patients in South Africa during the COVID-19 omicron wave compared with previous waves. JAMA.

[3] Suthar AB, Wang J, Seffren V, Wiegand RE, Griffing S, Zell E (2022). Public health impact of covid-19 vaccines in the US: observational study. BMJ.

[4] (2023). Advisory board document “Projected Severe Illness, Fatality, and Hospitalization Rates in the Seventh Wave (revised). https://www.mhlw.go.jp/content/10900000/000964719.pdf.

[5] Sudore RL, Lum HD, You JJ (2017). Defining advance care planning for adults: a consensus definition from a multidisciplinary Delphi panel. J Pain Symptom Manage.

[6] (2023). Report on the survey on attitudes towards medical care in the final stage of life. https://www.mhlw.go.jp/toukei/list/saisyuiryo_a.html.

[7] Nakanishi M, Nakashima T, Miyamoto Y, Yamasaki S, Nishida A (2022). Family caregivers' concerns about advance care planning for home-dwelling people with dementia: a cross-sectional observational study in Japan. BMC Palliat Care.

[8] Takazono T, Imamura Y, Kawakami K (2020). Discrepancies in preferences regarding the care of terminal-phase pneumonia in elderly patients among patients, families, and doctors: a multicenter questionnaire survey in Nagasaki, Japan. Respir Investig.

[9] Dassel KB, Towsley GL, Utz RL (2021). A limited opportunity: COVID-19 and promotion of advance care planning. Palliat Med Rep.

[10] (2023). A guide to the treatment of new coronavirus infections (COVID-19). https://www.mhlw.go.jp/content/000936655.pdf.

[11] (2023). US CDC. Science brief: evidence used to update the list of underlying medical conditions associated with higher risk for severe COVID-19. https://www.cdc.gov/coronavirus/2019-ncov/hcp/clinical-care/underlyingconditions.html.

[12] Barnato AE, Johnson GR, Birkmeyer JD, Skinner JS, O'Malley AJ, Birkmeyer NJ (2022). Advance care planning and treatment intensity before death among black, hispanic, and white patients hospitalized with COVID-19. J Gen Intern Med.

[13] Sun F, Lipinsky DeGette R, Cummings EC (2022). Capturing what matters: a retrospective observational study of advance care planning documentation at an academic medical center during the COVID-19 pandemic. Palliat Med.

[14] Omomo M, Tsuruwaka M (2018). Factors promoting ACP and factors hindering ACP: an analysis of the care process and specific support of home care nurses for elderly people living alone. J Japan Assoc Bioethics.

[15] Cousins E, Preston N, Doherty J (2022). Implementing and evaluating online advance care planning training in UK nursing homes during COVID-19: findings from the Necessary Discussions multi-site case study project. BMC Geriatr.

[16] Ye P, Fry L, Champion JD (2021). Changes in advance care planning for nursing home residents during the COVID-19 pandemic. J Am Med Dir Assoc.

[17] Asakawa S, Death AH (2020). Difference between western contries and Japan. Jpn J Fam Sociol.

